# Self-cloning of the Catalase Gene in Environmental Isolates Improves Their Colony-forming Abilities on Agar Media

**DOI:** 10.1264/jsme2.ME23006

**Published:** 2023-06-10

**Authors:** Motoyuki Watanabe, Kensuke Igarashi, Souichiro Kato, Yoichi Kamagata, Wataru Kitagawa

**Affiliations:** 1 Graduate School of Agriculture, Hokkaido University, Kita-9 Nishi-9, Kita-ku, Sapporo 060–8589, Japan; 2 Graduate School of Global Food Resources, Hokkaido University, Kita-9 Nishi-9, Kita-ku, Sapporo 060–8589, Japan; 3 Bioproduction Research Institute, National Institute of Advanced Industrial and Technology (AIST), 2–17–2–1, Tsukisamu-Higashi, Toyohira Ward, Sapporo 062–8517, Japan; 4 Bioproduction Research Institute, National Institute of Advanced Industrial and Technology (AIST), 1–1–1 Higashi, Tsukuba 305–8567, Japan

**Keywords:** hydrogen peroxide, agar plate, catalase, self-cloning, colony formation

## Abstract

Hydrogen peroxide (H_2_O_2_) inhibits microbial growth at a specific concentration. However, we previously isolated two environmental bacterial strains that exhibited sensitivity to a lower H_2_O_2_ concentration in agar plates. Putative catalase genes, which degrade H_2_O_2_, were detected in their genomes. We herein elucidated the characteristics of these putative genes and their products using a self-cloning technique. The products of the cloned genes were identified as functional catalases. The up-regulation of their expression increased the colony-forming ability of host cells under H_2_O_2_ pressure. The present results demonstrated high sensitivity to H_2_O_2_ even in microbes possessing functional catalase genes.

Two *Comamonadaceae* bacteria, *Rhodoferax* sp. OS-1 and *Curvibacter* sp. OS-4 were aerobically isolated from freshwater samples as microbes that were highly sensitive to H_2_O_2_-embedded agar medium (Watanabe *et al.*, 2022). Their colony-forming ability on agar medium was completely inhibited in the presence of 7.2‍ ‍μM H_2_O_2_ (Watanabe *et al.*, 2022). Common agar growth medium contains approximately 15‍ ‍μM H_2_O_2_ (Tanaka *et al.*, 2014). This concentration does not affect the growth of common laboratory strains, such as *Escherichia coli*, *Pseudomonas putida*, and *Bacillus subtilis*, but was shown to markedly inhibit the growth of environmental microbes, including OS-1 and OS-4 (Watanabe *et al.*, 2022).

H_2_O_2_ is endogenously generated by aerobic microbes during aerobic respiration, and is produced by *E. coli* at a rate of 10–15‍ ‍μM s^–1^ (Seaver and Imlay, 2001). Intracellular H_2_O_2_ is immediately degraded by various enzymes, including catalase (Seaver and Imlay, 2001; Mishra and Imlay, 2012; Sen and Imlay, 2021), because the accumulation of 0.5‍ ‍μM of intracellular H_2_O_2_ inhibits microbial growth (Sen and Imlay, 2021). Strains OS-1 and OS-4 both grow aerobically, and a genome sequencing ana­lysis of these strains revealed that they possessed at least one putative catalase gene despite exhibiting high sensitivity to H_2_O_2_ (Watanabe *et al.*, 2023). We hypothesized that if the putative catalase genes in OS-1 and OS-4 were properly expressed and translated products were functional, the colony-forming ability of both strains on agar media may be enhanced. However, the functions of these genes and their products remain unclear. High sensitivity to H_2_O_2_ may occur if (1) the putative catalase gene is a structural pseudogene with critical mutations (*e.g.*, a frameshift mutation) that hinder the formation of the proper amino acid sequence, (2) putative catalase is not expressed in the gene or its expression is insufficient to detoxify H_2_O_2_ from agar medium, or (3) the translated product does not function as a catalase enzyme. The colony-forming abilities of mutant strains of *Escherichia*, *Salmonella*, *Shewanella*, and *Yersinia* species lacking genes for the degradation of H_2_O_2_ were previously shown to be reduced (Ezraty *et al.*, 2014; Shi *et al.*, 2015; Wan *et al.*, 2017; Li and Imlay, 2018; Wan *et al.*, 2021).

The present study investigated the sequences and expression of catalase genes in H_2_O_2_-sensitive environmental strains. These genes were cloned and examined to confirm gene functionality and elucidate the relationship between catalase activity and colony-forming ability on artificial agar media.

Since both strains are highly sensitive to H_2_O_2_, their putative catalase genes were initially suspected to be structur­al‍ ‍pseudogenes; therefore, these genes were re-sequenced and their adequacy was analyzed. Based on a genome sequencing ana­lysis (Watanabe *et al.*, 2023), strain OS-1 was found to possess two putative catalase genes (locus tags of os1_07570 and os1_37390), while strain OS-4 harbored one gene (locus tag of os4_08060). Putative catalase genes in the OS-1 and OS-4 genomes were intact open reading frames (ORF) with proper start and stop codons, and no frameshift mutations were detected. The predicted amino acid sequences of these enzymes showed sequence similarities to previously reported catalases; these genes were less likely to be structural pseudogenes. A BLASTp (NCBI) ana­lysis revealed that the highest amino acid sequence identity of os1_07570 was 83.72% to the putative catalase gene of *Rhodoferax* sp. OV413, that of os1_37390 was 91.55% to the putative catalase gene of *Rhodoferax* sp. OV413, and that of os4_08060 was 96.19% to the putative catalase gene of *Rhodoferax aquaticus*. The amino acid sequences of these genes and the catalase genes with previously reported activities (*e.g.*, *E. coli* catalases) were utilized to construct the phylogenetic tree shown in Fig. 1. Each of the catalases from both strains showed the closest relationship with those identified in the genomes of *Rhodoferax* spp.; however, the enzymatic activities of the putative catalases of *Rhodoferax* spp. were not experimentally confirmed. In the tree, one OS-1 catalase (os1_37390) and OS-4 catalase (os4_08060) clustered with KatG enzymes that encode bifunctional catalase-peroxidase. Another OS-1 catalase (os1_07570) clustered with KatE enzymes that encode monofunctional catalases. Therefore, we designated these catalase genes as *katG_os1_* and *katE _os1_* for strain OS-1 catalases and *katG_os4_* for strain OS-4 catalase. The expression of these catalase genes was evaluated using a reverse transcription polymerase chain reaction (PCR). Their expression was not confirmed in wild-type OS-1 or OS-4 colonies, indicating that neither of these genes were expressed or their expression was below the detection level of the method used (data not shown).

Catalase genes were cloned to confirm their enzymatic activities. The putative catalase genes of OS-1 and OS-4 were amplified from the genomic DNA of OS-1 or OS-4 using PrimeSTAR GXL DNA Polymerase (Takara) with the following primers:

*katG_os1_*: forward primer; 5′-AGGAGACATTACATATGACCACCGAAGCCAAATGC-3′, reverse primer; 5′-CCTTGGATCCCTCGAGTTACGCCAGATCGAACCG-3′,

*katE_os1_*: forward primer; 5′-AGGAGACATTACATATGACCCCCACCGCTACCCAA-3′, reverse primer; 5′-CCTTGGATCCCTCGAGCTATGCAGGTACAGTGGC-3′, and

*katG_os4_*: forward primer; 5′-AGGAGACATTACATATGACTACTGAAGCCAAATGCC-3′, reverse primer; 5′-CCTTGGATCCCTCGAGTCACACGAGGTCGAAGCG-3′. PCR conditions were as follows: the initial denaturation of DNA at 94°C for 1‍ ‍min, followed by 30 cycles at 98°C for 10‍ ‍s and at 68°C for 1‍ ‍min kb^–1^, with a final extension at 68°C for 3‍ ‍min. Only the protein-coding region of each gene was amplified and gene fragments were separately inserted into a plasmid vector using an In-Fusion cloning kit (Takara), followed by the confirmation of “PCR error-free” by sequencing. Since the environmental isolates OS-1 and OS-4 have no previously established host–vector system, the broad-host-range vector pHRP308 (RSF1010 replicon) (Parales and Harwood, 1993) was used with the following modifications. The gentamycin-selective marker gene of pHRP308 was replaced with a kanamycin marker gene, and the *lacZ* gene was removed, yielding the vector, pCPKM. The plasmids with catalase gene inserts were named pOS1katG (harboring *katG_os1_*), pOS1katE (harboring *katE_os1_*), and pOS4katG (harboring *katG_os4_*). In addition, functional catalase genes from *E. coli* were used as a positive control (Loewen *et al.*, 1993; Singh *et al.*, 2008). The *katG* and *katE* genes were PCR-amplified from the genomic DNA of *E. coli* K-12, with the same PCR conditions as the amplification of other catalases, but using the following primers:

*katG*: forward primer; 5′-GCTAAGGAAGCTAAAATGAGCACGTCAGACGATATC-3′, reverse primer; 5′-TATGTTGCGACATTACAGCAGGTCGAAACGG-3′ and

*katE*: forward primer; 5′-CGACCTGCTGTAATGTCGCAACATAACGAAAAGAAC-3′, reverse primer; 5′-ACTGCCTTAAAAAAATCAGGCAGGAATTTTGTCAATC-3′.

The amplified *katG* and *katE* gene fragments were tandemly connected and inserted into the pCPKM vector, and the plasmid was named pECkatGE. To enhance the probability of gene expression, a bacterial consensus promoter (TTGACA-17 nt-TATAAT) and Shine-Dalgarno sequence (AGGAGA) were located upstream of the cloned genes on each plasmid (Fig. S1). These plasmids were introduced into strains OS-1 and OS-4 by electroporation. Electrocompetent cells were prepared as follows: OS-1 and OS-4 cultured in liquid peptone-yeast-glucose (PYG) medium (Tanaka *et al.*, 2014) were collected by centrifugation (1,500×*g* at 4°C), cell pellets were washed twice with sterilized 10% glycerol, and cells were finally re-suspended in 10% glycerol. Using the Gene Pulser Xcell Electroporation System with the following parameters: exponential protocol, voltage: 1–1.6‍ ‍kV, resistance: 400‍ ‍Ω (Bio-Rad), the pOS1katG and pOS1katE plasmids were introduced into OS-1 cells and pOS4katG into OS-4 cells, while pECkatGE (positive control) and the blank vector pCPKM (negative control) were introduced into OS-1 and OS-4 cells.

To confirm cellular catalase activity, the wild-type and transformed strains of OS-1 and OS-4 were examined using the conventional catalase test. Bacterial colonies grown aerobically on solid medium were transferred onto a glass slide, and 30% commercially available H_2_O_2_ was dropped on the top. The degradation of H_2_O_2_ was assessed by observing the formation of oxygen bubbles (Fig. 2). Wild-type OS-1 and OS-4 and their blank vector transformants did not form bubbles (Fig. 2A, B, F, and G), whereas the transformants harboring cloned catalase genes rapidly formed bubbles after the application of H_2_O_2_ droplets (Fig. 2C, D, E, H, and I).

These results indicate that the broad-host-range vector used was successfully maintained and also that *E. coli* catalase was functionally expressed in these strains (Fig. 2C and H). Furthermore, the results of self-cloning experiments demonstrated that the catalases produced in OS-1 and OS-4 were functionally active enzymes (Fig. 2D, E, and I).

The colony-forming abilities of transformed OS-1 and OS-4 were examined by cultivating them on agar medium. Wild-type strains and their transformants were initially cultured in PYG liquid medium and diluted until OD_600_ reached 0.1, followed by a 10-fold serial dilution to 10^–7^ in sterile distilled water. After each dilution, 10‍ ‍μL was spotted on the surface of PYG solid medium embedded with different H_2_O_2_ concentrations. The preparation of solid medium and the measurement of H_2_O_2_ concentrations were performed as described in our previous study (Watanabe *et al.*, 2022). After an incubation at 20°C for 6 days, the growth of each strain was evaluated. On the plate containing 0.9‍ ‍μM H_2_O_2_, strains OS-1 and OS-4 both showed similar growth among wild-type, blank vector, and catalase gene-containing transformants up to the 10^–6^ or 10^–5^ dilution (Fig. 3A). Regarding strains OS-1 and OS-4, the growth of wild-type and blank vector transformants on the plate with 13.3‍ ‍μM H_2_O_2_ was only observed on the first spot. *E. coli* catalase transformants exhibited growth up to the 10^–3^ dilution spot, and self-cloning transformants showed growth up to the 10^–5^ dilution spot (Fig. 3B). The colony-forming units (CFU) of wild-type strains growing on the agar plate containing 13.3‍ ‍μM H_2_O_2_ decreased to approximately 10^–5^ order of magnitude compared to the CFU of strains growing on the agar plate containing 0.9‍ ‍μM H_2_O_2_. Self-cloning transformants maintained the original CFU count even in the presence of 13.3‍ ‍μM H_2_O_2_. Furthermore, the original CFU count of the self-cloning transformants was maintained in the presence of 47.9‍ ‍μM H_2_O_2_ (data not shown). Collectively, these results demonstrated that endogenous catalase expression and activity were crucial for colonization.

Although putative catalase genes were identified in the genomes of OS-1 and OS-4, their mRNA expression was not detected. To elucidate the function of the gene products, we self-cloned these catalase genes. The present study is the first to successfully self-clone the catalase genes of environmental H_2_O_2_-highly sensitive isolates using a broad-host-range plasmid vector, and we demonstrated that the products expressed were functional catalases. In addition, the results obtained herein indicate that the function and activity of a gene cannot be predicted by its presence in the genome, even for well-known and studied genes. Since the up-regulated expression of self-cloned catalase genes markedly increased the colony-forming abilities of the two strains (Fig. 3), we showed, for the first time, that H_2_O_2_ in the agar plate was the main component that inhibited colony formation by strains OS-1 and OS-4, and the insufficient expression of the catalase genes was one of the causes of their high sensitivity to H_2_O_2_. A promoter search ana­lysis by GENETYX-MAC v.21.0.1 software (GENETYX CORPORATION) revealed the presence of putative promoter and Shine-Dalgarno sequences at the upstream regions of each catalase gene in the OS-1 and OS-4 genomes. These native promoters scored 40 to 50 by the software, while the bacterial consensus promoter used in the present study scored 77. Although native promoter strength and regulation in wild-type OS-1 and OS-4 remain unknown, the consensus promoter may have markedly up-regulated catalase gene expression in the recombinant strains. The present results imply the presence of numerous similar environmental microbes that are highly sensitive to H_2_O_2_ in the agar plate, regardless of whether functional catalase genes were present in their genomes. These microbes may be able to tolerate the H_2_O_2_ pressure found in natural environments, but may be vulnerable to the amount of H_2_O_2_ in agar medium. H_2_O_2_ concentrations detected in a natural environment are always in the nanomolar order (Wilson *et al.*, 2000; Takeda *et al.*, 2018; Garcia *et al.*, 2020), which may not be a threatening concentration as exogenous H_2_O_2_; therefore, for many microbes, the expression of catalase genes may not be required under natural conditions. The presence of environmental microbes possessing the enzyme that decomposes H_2_O_2_ concentrations in the micromolar order is of interest; however, the expression of these genes may not be effectively induced on agar plates.

To decrease H_2_O_2_ concentrations in agar media, previous studies applied 20–100 units mL^–1^ bovine liver catalase enzyme (Olson *et al.*, 2000; Kawasaki and Kamagata, 2017; Adam *et al.*, 2018), which successfully increased the colony yield of environmental microbes; however, the routine preparation of this medium is expensive. Moreover, the presence of other growth inhibitors in the agar plate has been reported (Kawasaki and Kamagata, 2017), and their degradation may require more than the simple supplementation of inexpensive enzymes. Under these conditions, the potential detoxifying abilities of the genomes of microbes may enhance their colony formation.

Although the catalase gene cloning technique used in the present study significantly improved the colony-forming abilities of the OS-1 and OS-4 strains, this technique is not always easily applied to a number of environmental microbes because host–vector systems and gene introduction methods strongly depend on and differ among strains. A novel pre-treatment or modified cultivation technique that effectively induces the expression of catalase genes (or other functional genes that degrade other growth inhibitors) in each microorganism is needed to improve colony-forming abilities, thereby increasing opportunities to isolate novel environmental microbes.

## Citation

Watanabe, M., Igarashi, K., Kato, S., Kamagata, Y., and Kitagawa, W. (2023) Self-cloning of the Catalase Gene in Environmental Isolates Improves Their Colony-forming Abilities on Agar Media. *Microbes Environ ***38**: ME23006.

https://doi.org/10.1264/jsme2.ME23006

## Supplementary Material

Supplementary Material

## Figures and Tables

**Fig. 1.**
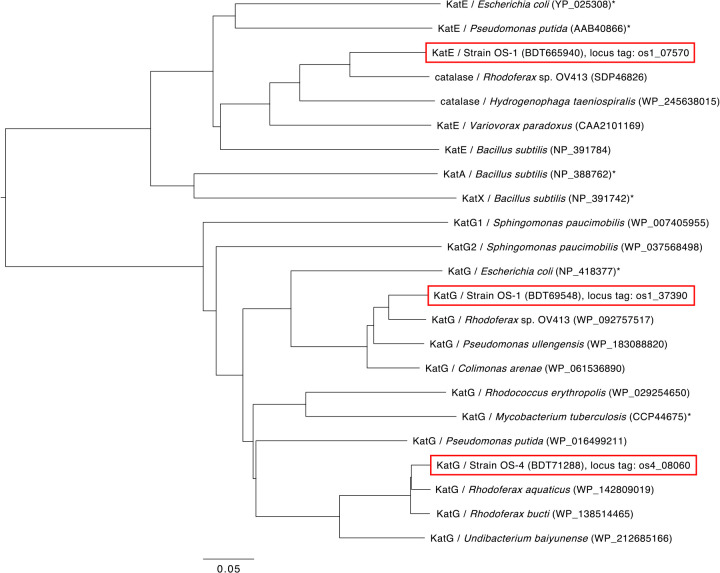
Phylogenetic tree of catalases based on their predicted amino acid sequences. The catalases present in OS-1 and OS-4 are shown in the red frame. Catalases experimentally tested for their activities are marked with an asterisk “*”. The scale bar indicates 0.05 substitutions per amino acid position. Protein ID numbers are shown in parentheses after the host name.

**Fig. 2.**
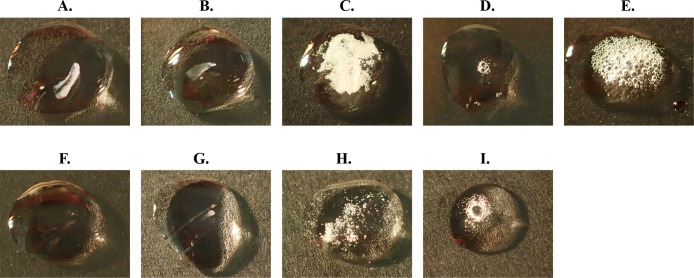
Detection of cellular catalase activities of OS-1, OS-4, and their transformants. Strains used in the panels; wild-type OS-1 (A), OS-1+pCPKM (B), OS1+pECkatGE (C), OS-1+pOS1katG (D), OS-1+pOS1katE (E), Wild-type OS-4 (F), OS-4+pCPKM (G), OS-4+pECkatGE (H), and OS-4+pOS4katG (I). Images were taken right after 20‍ ‍μL of 30% H_2_O_2_ was dropped on the top of smeared colonies.

**Fig. 3.**
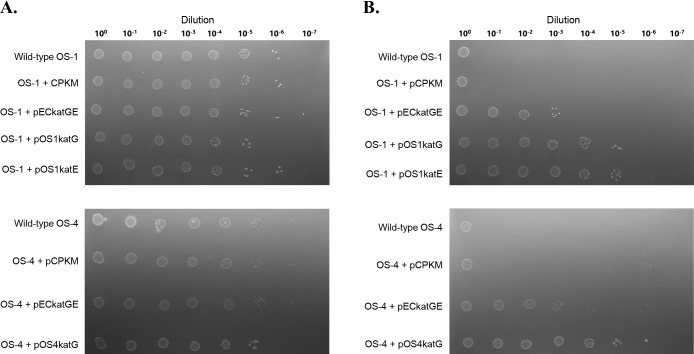
Growth of OS-1, OS-4, and their transformants on PYG agar plates containing 0.9‍ ‍μM (A) or 13.3‍ ‍μM (B) H_2_O_2_. Strains grown aerobically in PYG liquid medium were collected and re-suspended to obtain an OD_600_ of 0.1 (dilution 10^0^), and the cell suspension was serially diluted and 10‍ ‍μL of each dilution was spotted. Images were taken after an incubation at 20°C for 6 days.
