# Supplementary material for: Self-cloning of the Catalase Gene in Environmental Isolates Improves Their Colony-forming Abilities on Agar Media
Source: Microbes Environ. 2023 Jun 10;38(2):ME23006. doi: 10.1264/jsme2.ME23006 (PMC10308234; doi:10.1264/jsme2.ME23006)
Supplement: Supplementary file 1 — Supplementary Material [file 38_23006_s1.pdf]

Supplemental Material

Self-cloning of catalase gene in environmental isolates improves their colony formation abilities on agar media

Motoyuki Watanabe, Kensuke Igarashi, Souichiro Kato, Yoichi Kamagata and Wataru Kitagawa

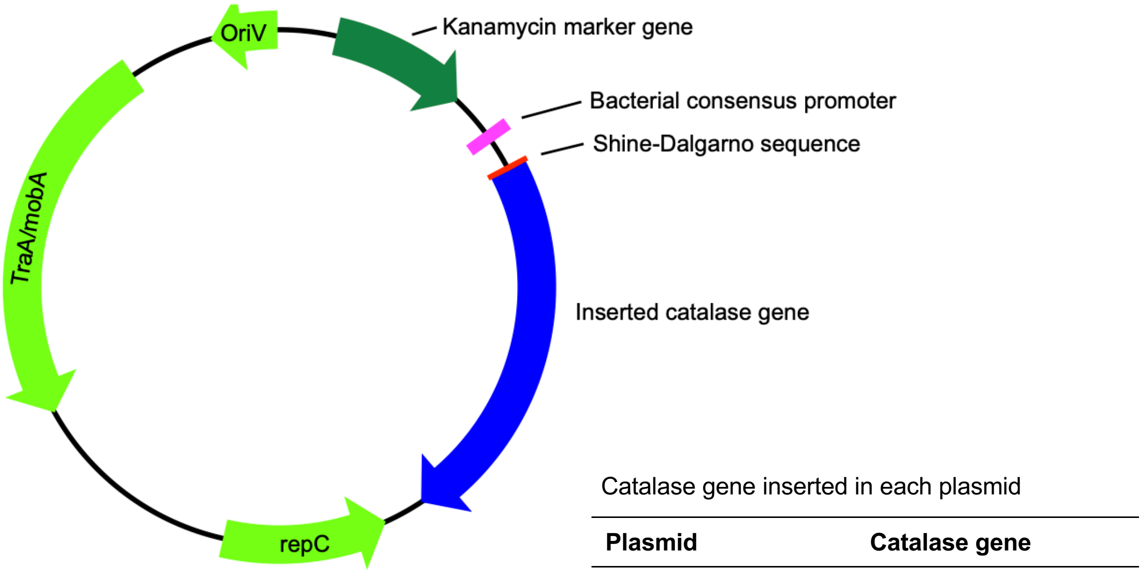

Catalase gene inserted in each plasmid

**Plasmid**	**Catalase gene**
pCPKM (Blank vector)	None
pOS1katG	katG_{OS1}
pOS1katE	katE_{OS1}
pOS4katG	katG_{OS4}
pECkatGE	$\mathit{katG}_{E.coli}$, $\mathit{katE}_{E.coli}$

Fig. S1. Gene map of the plasmids constructed in this experiment. Plasmids were contructed based on the plasmid vector pCPKM, which has rsf1010 replicon.
